# Helping cancer patients quit smoking by increasing their risk perception: a study protocol of a cluster randomized controlled trial

**DOI:** 10.1186/s12885-015-1496-2

**Published:** 2015-06-30

**Authors:** William H.C. Li, Sophia S.C. Chan, Kelvin M. P. Wang, T.H. Lam

**Affiliations:** 1 School of Nursing, The University of Hong Kong, 4/F, William MW Mong Block, No 21 Sassoon Road, Pokfulam, Hong Kong; 2 School of Public Health, 5/F, William MW Mong Block, No 21 Sassoon Road, Pokfulam, Hong Kong

**Keywords:** Cancer, Quality of life, Risk communication, Smokers, Smoking cessation

## Abstract

**Background:**

Despite smoking cessation can largely improve cancer prognosis and quality of life, many patients continued smoking after the diagnosis of cancer. This study aims to test the effectiveness of a smoking cessation intervention using risk communication approach to help cancer patients quit smoking, and to improve their health related quality of life.

**Methods:**

A cluster randomized controlled trial will be employed. Cancer patients who continued smoking after the diagnosis of cancer and have medical follow-up at the out-patient clinics of the five acute hospitals in Hong Kong will be invited to participate. Subjects in the experimental group will receive (1) health warnings of smoking based on a special designed leaflet; and (2) a patient-centred counseling from nurse counselors with emphasis on risk perceptions of smoking to cancer prognosis. Additionally, they will receive two more telephone counseling at 1-week and 1-month. Control group receive standard care and a generic self-help smoking cessation booklet. Outcomes measure include (a) self-reported and the biochemically validated quit rate, (b) patient’s smoking reduction by at least 50 % compared to baseline, (c) quit attempt(s), (d) change in the intention to quit, (e) change in risk perceptions of smoking, and (f) change in health related quality of life.

**Discussion:**

This study will make an important contribution to evidence-based practice by testing the effectiveness of a tailored smoking cessation intervention for cancer patients. The results will support the development of clinical practice guidelines to promote smoking cessation in cancer patients to improve their prognosis and quality of life.

**Trial registration:**

ClinicalTrials.gov NCT01685723. Registered 9 November 2012.

## Background

Smoking is the most significant preventable cause of cancer in the world [1]. Research indicates that current smokers have a twofold to threefold increased risk of cancer, and about 90 % of lung cancers are attributed to smoking [2, 3]. Apart from lung cancer, cigarette smoking can cause many different types of cancer including cancer of the oropharynx, larynx, oesophagus, trachea, bronchus, acute myeloid leukaemia, stomach, liver, pancreas, kidney, ureter, and bladder, and colorectal [2].

Recent advances in medical technology have dramatically improved the survival rate for most types of cancer. However, cancer patients who continue to smoke are at greater risk for second primary, cancer recurrence, and all causes of mortality [4]. Furthermore, cigarette smoking can not only reduce the effectiveness of medical treatments for cancer including radio- and chemo-therapies [5, 6], but also increase the risk of therapy-related side-effects [7]. Conversely, there is strong evidence that quitting smoking after being diagnosed with cancer could reduce the risk of disease advancement [8], minimize adverse treatment-related effects, improve prognosis, and enhance the quality of life [9]. Given the beneficial effects of cigarette smoking cessation and the hazardous effects of continued smoking on cancer patients’ physical and psychological health, it is of paramount importance for healthcare professionals to help this vulnerable group to quit smoking [10].

Medical attention at out-patient clinics of smokers who had been diagnosed with cancer can be developed as an excellent “teachable model” as it provides an invaluable opportunity for them to initiate smoking cessation to improve their health. It also presented healthcare professionals with a golden opportunity to advise smokers to quit while they are waiting for medical consultation or follow-up. Nevertheless, cigarette smoking is addictive and quitting is very difficult, with a high rate of relapse, particularly among those chronic patients with high nicotine dependency [11, 12]. Previous studies showed that 47 % smokers with lung cancer in USA and about one third of cancer patients in western countries continued to smoke after receiving a cancer diagnosis [4, 13–15].

In a recent qualitative study [16] investigating the risk perceptions, and the behaviour, attitudes, and experiences of Chinese current smokers and ex-smokers who have been diagnosed with cancer, the results reveal that many of the informants had inadequate knowledge of the association between smoking and cancer. Besides, many informants who continued smoking were unaware of the beneficial effects of quitting, including ameliorating adverse effects of treatment and improving prognosis and quality of life. Furthermore, many informants had misconceptions that a moderate amount, such as half a pack of cigarettes per day, was not detrimental to their physical health. Some even believed that quitting would harm their physical well-being as their bodies had become desensitized to the chemicals in tobacco after long-term smoking. Additionally, some informants were worried about losing the “psychological benefits” of smoking once they quit and thus perceived barriers to quit outweighed the perceived benefits. The findings of this study [16] address an important yet under-researched area, as very few smoking cessation programs targeting cancer patients, and only very few healthcare professionals help this vulnerable group to quit smoking [17]. There is an imperative need for healthcare professionals to design special interventions that use strong warning to clearly communicate the risk of continued smoking to this group as a strategy to enhance their motivation to quit. The findings of the study [16] provide a guide for developing an effective intervention protocol that will demystify the misconceptions about smoking among these vulnerable individuals and increase their perception of the risks of continued smoking and benefits of quitting.

### Current study

The present study aims to test the effectiveness of a specially designed smoking cessation intervention using a risk communication approach for cancer patients who are smokers, to (i) achieve a higher quit rate, (ii) improve their smoking behaviour, (iii) motivate their intention to quit smoking, (iv) improve their risk perceptions of continued smoking, and (v) improve the quality of life.

### Conceptual framework

**Fig. 1:**
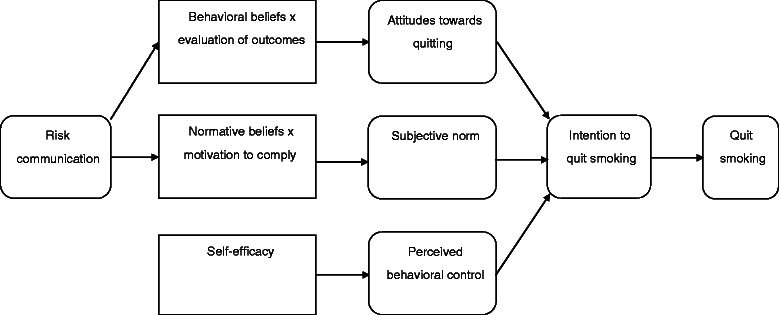
Theoretical framework – a modified Theory of Planned Behavior (TPB) model

The theoretical framework is shown in Fig. 1. The framework is constructed with reference to the nicotine addiction context in smoking [18], and a modified Theory of Planned Behaviour (TPB) to guide the development of the smoking cessation intervention [19]. We assume smokers who have cancers will quit smoking if they have positive attitudes, subjective norms and perceived control on quitting, while the antecedents are mediated by intention. In addition, we will use a risk communication approach in the Self-Regulation Model of Illness [20] to influence patients’ attitudes toward quitting (behavioural beliefs) and their normative beliefs. We will apply part of the Transtheoretical Model (TTM) to guide the risk communication (in form of the pros and cons of behavioural change) and increase the self-efficacy of participants [21]. The concept of risk perceptions have been found to be a cue to promote healthy behaviour among patients.

## Methods/Design

**Fig. 2:**
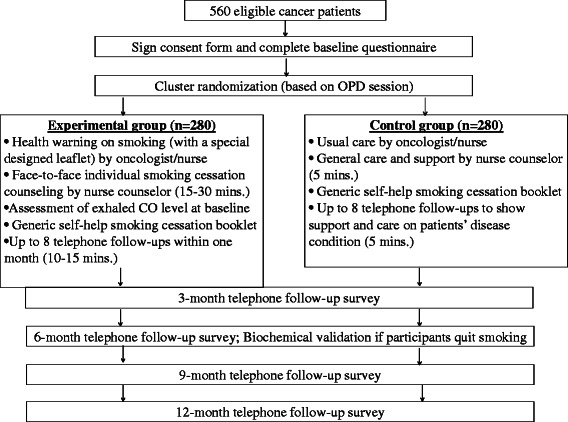
Study protocol (CONSORT diagram)

The study design is shown in Fig. 2. It is a cluster randomized controlled trial to test the effectiveness of a smoking cessation intervention using risk communication approach to help cancer patients quit smoking. The settings will be the oncology out-patient clinics of the five acute hospitals in Hong Kong.

### Study sample and recruitment

Cancer patients fulfilling the following inclusion and exclusion criteria will be invited to participate in the study. The inclusion criteria are: (1) smoked weekly in the past 6 months, (2) diagnosed with cancer that are related to smoking [2], such as cancer of the oropharynx, larynx, lung, oesophagus, trachea, bronchus, acute myeloid leukaemia, stomach, liver, pancreas, kidney, ureter, and bladder, and colorectal, (3) diagnosed with cancer for at least 6 months (such that their conditions and treatments would be stable), (4) patients in all stages I, II, III, or IV, (5) aged 18 or above, and (6) can communicate in Cantonese. The exclusion criteria are: (1) those with unstable medical conditions as advised by the doctor in charge, (2) poor cognitive state, (3) having mental illness, and (4) those participating in other smoking cessation program. The oncologists and oncology nurses will assess and inform us the stability and suitability of the cancer patient to participate. The patients would be at different phases/ stages but they will also be eligible, if confirmed by the oncologists/ oncology nurses.

### Procedure

Approval for the study has been obtained from five hospital ethics committees (Kowloon West Cluster Research Committee; Hong Kong East Cluster Ethics Committee; Kowloon Central/Kowloon East Research Ethics Committee; Hong Kong West Cluster Ethics Committee; and New Territories West Cluster Clinical & Research Ethics Committee). To ensure the rights of all participants will be protected, the research carried out will be in compliance with the Helsinki Declaration (http://www.wma.net/en/30publications/10policies/b3/index.html). Research assistants will approach patients in the oncology out-patient clinic and ask them whether they are smokers or not. The eligible subjects will then invite to participate in this study after they are told the purpose of the study. They will be given the option of participating or refusing involvement in the study and will be told that their participation is voluntary without prejudice. Written consent will then obtain from all participants.

To evaluate the effectiveness of a smoking cessation intervention using risk communication approach, a total of 560 eligible Chinese cancer patients were recruited from five out-patient clinics in Hong Kong. After completing the baseline questionnaire, subjects will receive proposed intervention according to their group assignment. Four consecutive (3-, 6-, 9- and 12-month) follow ups will be conducted by trained interviewers (blinded to the group assignment) with all subjects via telephone. We will assess hospital readmission and other clinical outcomes from the participants’ medical records accordingly. Patients who have successfully quit smoking at 6-month will be invited to come back to the out-patient clinic and have biochemical validation tests (saliva cotinine test and exhaled CO test). We will offer HKD300 (USD38.5) per client to cover their travel expenses and time cost. From our previous experience, such an incentive is necessary to secure a sufficiently high response rate.

#### Training and quality assurance of the nurse counsellors and research assistants

All the research nurses are qualified smoking cessation counsellors, and they will be provided with specific training workshop by the principal and co-investigators prior to the commencement of the study. They will be equipped with the necessary knowledge and skills to deliver both the smoking cessation counselling using the motivational interviewing techniques and stages of change model. Regular case conference, quality checks through audio-taping, and audit procedures will be conducted to ensure and maintain the quality and uniformity of the counselling interventions. The research assistants will be trained by the principal and co-investigators to screen eligible case, conduct baseline and follow-up surveys and procedures of biochemical validations (saliva cotinine test and exhale CO test). Briefing sessions will also be provided to oncologists and oncology nurses in using the special designed leaflets.

Since the same batch of oncologists and oncology nurses working in the setting would contact cancer patients in both the intervention and control group, a quality assurance mechanism will be used to prevent the contamination of intervention and control group: (1) a clear instruction and reminder will be given to the participating doctors and nurses before the start of the RCT study, and we will explain the principle of RCT and reinforce them the different treatments for the experimental and control groups; (2) a specific risk communication leaflet will be attached in front of the patients’ medical records in the intervention sessions, and doctors and nurses will fill up a checklist to make sure if they have delivered the intervention at the end of each consultation; (3) patients in the experimental group will be invited to complete a satisfaction survey during telephone follow-up to see if they have received the risk communication messages from physicians and nurses during baseline intervention; and (4) doctors and nurses will be reminded that they need not deliver the intervention during the “control” clinic sessions and only usual care will be given.

#### Quality and data security control

This study will follow the protocol of quality assurance developed by a previous project [22]. The principal and co-investigators, project coordinator and nurse counsellors will set up orientation meetings with oncologists, nurse managers and frontier nurses to explain the protocol, the flow of logistics and examine the physical facilities available in the hospital. The principal and co-investigators will answer any queries raised by oncologists and nurses immediately at any time point. The project coordinator will be present during the first week to monitor the subject recruitment and data collection process. All blank and completed instruments will be sealed in separate opaque envelopes, which will be kept in a locker with keys provided by hospitals, while the project coordinator will collect the filled instruments weekly by hand. All collected instruments will be saved in a locker with keys in the institution of the principal investigator. The project coordinator and other research assistants of this project will be responsible to input the data into SPSS, with the dataset encrypted in an assigned personal computer.

### Randomisation

Randomization will be done with each session in an out-patient clinic as a cluster unit. Currently there are 10 sessions per week per study site. Before the study, we will randomly draw the sessions by computer generated random sequence (block randomization with each hospital as a block unit), so that 5 sessions will be treated as intervention group and another 5 sessions will be treated as control group per week. Participants who consent to participate in the study will be assigned to either intervention or control group depending on the out-patient clinic session that they are seeking consultation. By using this experimental design, we can ensure similar proportion of smoking cancer patients will fall into the intervention and control group in each out-patient clinic, and each out-patient clinic will have a similar proportion of cancer patients into the intervention/ control group. We expect the intervention and control group would have similar socio-demographic profile, smoking history, and clinical history and we shall check for any differences, and if any, we shall adjust for them in the analyses. In using the cluster randomization design with a session being a cluster unit, the patients who are randomized into the experimental and control groups would have less chance to meet each other in the out-patient clinic and discuss the contents of the smoking cessation interventions.

### Intervention

#### Control group

Subjects in the control group will receive usual care provided at the site, a generic self-help smoking cessation booklet published by the Hong Kong Council on Smoking and Health, a brief face-to-face counselling at baseline plus telephone follow ups by nurses to show support and care on their disease condition.

#### Experimental group

Apart from receiving a generic self-help smoking cessation booklet, subjects in the experimental group will receive a specifically designed risk communication leaflet from oncologists/ oncology nurses during the medical consultation. They will be warned the risk of continued smoking which can affect their cancer treatment and prognosis. This brief advice aims to increase the risk perceptions and normative beliefs of participants, hence affect their behavioral beliefs and subjective norms towards quitting. Furthermore, subjects will receive a patient-centered motivational intervention by an experienced nurse counsellor focusing on: (1) risk communication based on self-regulation model of illness for cancer patients; (2) the stage-matched smoking cessation intervention (Table 1). The risk communication component focuses on the relationship of smoking and cancer diagnosis, treatment and prognosis as a trigger to think about quitting. The components are modified from another study to help lung cancer patients quit smoking, with a self-reported quit rate of 24 % at 6 months [20]. The stage-matched smoking cessation intervention aims to (1) increase awareness on the needs to quit smoking; (2) motivate and increase confidence in ability to quit; (3) set a quit plan and boost their self-efficacy to resist smoking; and (4) discuss possible withdrawal symptoms and relapse prevention strategies. The counselling process will take about 15–30 min. In addition, each subject will receive at least one boost up telephone intervention within one week and another telephone intervention within one month by nurse counsellor. The follow up intervention aims to assess the progress of their action plan and barriers encountered in the behavioral change process as well as to engage them in the process, enhance their self-efficacy, and identify individual barriers and facilitators. Each telephone counselling will take 10–15 min.

**Table 1:** Outline of smoking cessation intervention to be delivered by nurse counselor

At baseline
Stage-matched Smoking Cessation Counseling
(a) Pre-contemplation: Increase awareness of need to change; decisional balance; applying 5 “R”s: relevance, risks, rewards, roadblocks, repetition
(b) Contemplation: Motivate and increase confidence in ability to quit smoking; enhancing confidence in quitting by reinforcing achievement in previous quit attempt(s)
(c) Preparation: Set a quit plan and boost self-efficacy to resist smoking; discuss possible withdrawal symptoms and relapse prevention strategies
Risk communication via Self-regulation model of Illness
(a) Identity – recognizing the sign and symptoms of cancer
(b) Help patients understand the cause of cancer (smoking)
(c) Explain to patients the consequence of continue smoking or smoking cessation
(d) Emphasis the stage or cancer prognosis can be controlled by stopping smoking
(e) Reinforce patients’ abilities to change the overall timeline or prognosis of cancer if they can quit smoking substantially
At 1-week and 1-month telephone counseling
(1) Assess health-related lifestyle practices with emphasis on smoking cessation,
(2) Progress of patient’s action plan,
(3) Assess barriers encountered in the behavior change process,
(4) Boost self-efficacy, and
(5) Reinforce cancer related risk perceptions of continue smoking

### Measures

#### Structured questionnaire

A structured questionnaire will be developed by adopting or modifying international and/or locally validated instruments. The questionnaire gathers information including smoking and quitting history, risk perceptions of smoking [23], illness perception [24], intention to quit smoking (stage of readiness to quit) [21], antecedent factors of the TPB model (behavioral beliefs, outcome evaluations, normative beliefs, motivation to comply) [25], pros and cons of smoking (decisional balance), self-efficacy to resist smoking [26], quality of life (SF-12 v2) [27], other lifestyle risk factors (drinking, physical activities, and fruit and vegetable intake), demographic information such as age, gender, and marital status, and clinical information including height, body weight, body mass index, time to diagnose cancer, stage of cancer, number of tumor sites, blood pressure, and history of surgery, radiotherapy and chemotherapy. The demographic and clinical information will be obtained from the medical records.

### Process evaluation

Ten patients from the experimental group will be invited to participate in an in-depth interview after they have completed the 6-month follow up survey to examine their experience in quitting and the effect of the interventions from their perspective. Questions such as their satisfaction, perceptions on the advantages and disadvantages of the intervention, and any other suggestions for improvement will be explored and discussed

### Primary and secondary outcomes

The primary outcome is the self-reported 7-day point prevalence (PP) quit rate of the smoking cancer patients during 6-month follow up. The secondary outcomes include (a) self-reported 7-day PP quit rate at 12-month follow-up and the biochemically validated quit rate at 6-month follow-up (non-smoking status is confirmed by a carbon monoxide level in expired air < 9 ppm and saliva cotinine level < 115 ng/mL in parallel test), (b) percentage of patients reduced smoking by at least 50 % at 6- and 12-month follow-up compared to baseline, (c) percentage of patients with quit attempt(s) at 6- and 12-month follow-up, (d) change in the intention to quit smoking (stage of readiness to quit) at 6- and 12-month follow-up compared to baseline, (e) change in risk perceptions of continued smoking at 6- and 12-month follow-up compared to baseline, and (f) change in health-related quality of life at 6- and 12-month compared to baseline.

### Power calculation

Sample size is calculated by “Power Analysis and Sample Size (PASS) v. 13” (http://www.ncss.com/pass.html) and based on the main outcome variable according to the main hypothesis, the 7-day point prevalence self-reported quit rate at 6-month in the experimental group is higher than the control group. A previous RCT study [28] conducted in Australia, which applied motivational intervention to 137 smoking patients with mixed cancer sites. Based on that study, the self-reported 7-day point prevalence quit rate at 6-month follow up was 29 % for the intervention group and 18 % for the control group. To detect a statistical difference at 5 % significant level and a power of 0.8, 233 patients will be needed in each of the 2 groups (based on independent samples Fisher exact test). With reference to the local RCT on smoking cessation intervention for cardiac patients [22], we assume a 15 % attrition rate at the 6-month, and 274 patients will be required in each group to achieve a significant outcome. Accounting for the clustered randomized sampling design with 20 clusters (sessions) and an intra-class correlation of 0.002, 280 patients will be required per group, adding up the total sample size to 560 from the experimental and control groups together.

From the information provided by our clinical partners on site screening, there are around 2000 new patients per hospital per year. According to our previous qualitative study [23] conducted in an out-patient clinic on Chinese current smokers and ex-smokers, about 13.7 % patients continued smoking after receiving a cancer diagnosis (274: 13.7 % of 2,000). However, more than 50 % of these smokers were reluctant to quit (137: 50 % of 274). In a rough estimation, there will be around 700 eligible subjects in five acute Hospital Authority hospitals each year. We have confidence that we can recruit adequate subjects (560) according to the power analysis within an 18-month (78-week) recruitment period.

### Analysis

Data analysis was performed using the Statistical Package for Social Science software, version 20.0 for Windows. When assessing the effectiveness of interventions between the two groups, we will first compare the baseline characteristics of the patients using chi-square test for categorical variables and *t*-test or Wilcoxon rank-sum test for continuous variables between the experimental and control groups. The primary analysis will be an unadjusted intention-to-treat analysis of the difference in self-reported 7-day point prevalence quit rate of the smoking cancer patients during 6-month follow up between the two groups. The analysis will be performed using Pearson’s chi-square test or with the use of Fisher’s exact test, if there were five or fewer participants per cell. Similar approach will be used to estimate the differences in secondary outcomes (e.g. self-reported 7-day PP quit rate at 12-month follow-up and the biochemically validated quit rate at 6-month follow-up, percentage of patients reduced smoking by at least 50 % at 6- and 12-month follow-up compared to baseline, and quality of life at 6 and 12 months) between groups. Crude odds ratios (ORs) for quitting will be estimated using logistic regression model and were compared with ORs that were adjusted for baseline variables (e.g. sex, age, education, stage of readiness). Those who are lost to follow-up or refuse to participate in the validation tests, will be treated as smokers with no reduction in cigarette consumption compared with (a) baseline, as the main analysis (by intention to treat), (b) the most recent level and (c) complete case (per protocol) analysis by excluding subjects with missing data as a sensitivity analysis.

For the process evaluation, all qualitative data from interviews will be tape-recorded and transcribed verbatim. Content analysis will be employed and themes identified. The constant comparative method will be used to search for common constructs and themes about the implementation and the effect of the intervention as perceived by the participating subjects.

## Discussion

Smoking is the most significant preventable cause of death, causing six million deaths annually worldwide [1]. Although the prevalence of smoking has declined significantly over the last 50 years, smoking-attributable disease and death, in particular cancer have risen greatly [2]. Evidence shows that smoking cessation can largely improve cancer prognosis and quality of life among cancer patients. However, many cancer patients who continued smoking after the diagnosis often had inadequate knowledge of the association between smoking and cancer, as well as misconceptions about smoking. This study aims to test the effectiveness of a smoking cessation intervention using risk communication approach to help cancer patients quit smoking, and to improve their health related quality of life.

To the best of our knowledge, this is the first RCT that will conduct to help cancer patients quit smoking by increasing their risk perception. The originality and significance of the research question will address an important yet under-researched area, as healthcare professionals often underestimate the value of helping cancer patients quit smoking. It is anticipated that the results will motivate more healthcare professionals to help smokers who have been diagnosed with cancer quit smoking routinely in clinical and community settings. Meanwhile, it is crucial that healthcare professionals should be offered relevant training so as to enhance their self-efficacy and confidence in promoting smoking cessation to cancer patients.

### Implications for clinical practice

This study will make an important contribution to evidence-based practice by testing the effectiveness of a tailored smoking cessation intervention targeting cancer patients.

If it is proven to be effective, the findings of this research have great potential to influence practice not just in out-patient clinics but also in wider health service and other public sector settings. Most importantly, the results primarily serve the purpose to support the development of clinical practice guidelines and interventions to promote smoking cessation in cancer patients to improve their cancer prognosis and in long-term, increase their survival time and quality of life. For the 280 cancer patients in the experimental group, we expect to help at least 81 to quit smoking at 6-month (29 %). For the sessions that are randomized as the experimental group, all cancer patients (no matter they participate or not into the study) would receive a specially designed leaflet and health warning from oncologists/ nurses. In addition, all the 580 subjects would receive a generic self-help booklet to help them quit smoking. Among the quitters who have lung cancer, their risk of cancer recurrence can be reduced by 20–47 %; the mortality rate (all-cause) can be reduced by 46–66 %; the risk of second primary tumor can be reduced by 76 % (among those with limited stage small cell lung cancer); and the likelihood of 5-year survival can be doubled, compared to still smokers [16]. Patients with cancer in other sites will also have clinical benefits after they quit smoking. Subsequently, the quality of life among cancer patients who stopped smoking can also be improved.

### Potential limitations or barriers

There are serval potential limitations or barriers of implementing the proposed intervention in clinical practice. First, the proposed intervention is relatively comprehensive, which generally takes 30 to 45 min to implement including the baseline assessment. According to our previous smoking cessation projects in outpatient clinics, some patients are too impatient to undergo a long intervention and some are reluctant to participate for fear that they might miss or experience delays in their medical consultation or other medical procedures. Nevertheless, the intervention will only implement during the waiting time for medical consultation in the out-patient clinics and all potential subjects will be reinforced by nurse counsellors that they will not miss the medical consultation or medical procedures through participating in this study. Second, the intervention that we proposed for the experimental group is not on a one-off basis. Each subject will receive at least one boost up telephone intervention within one week and another telephone intervention within one month by a nurse counsellor. Such practice may not be feasible in busy clinical settings in Hong Kong at this moment. Nevertheless, such approach is necessary and crucial as smoking is addictive and quitting is very difficult, with a high rate of relapse. Based on our previous smoking cessation projects on promoting smoking cessation, continuous reminding and offering support is of paramount importance to achieve the ultimate goal of complete cessation. Indeed, each telephone counselling will take only 10–15 min. If the telephone follow up effect size is proven and substantial, this would justify additional resources for such practice. In the short run, smokers may be referred to smoking cessation hotline for continuous support and follow up after receiving the initial intervention by healthcare professionals. Third, we do not use biochemical indicator as the primary outcome, which is more scientific and objective to ascertain whether a person has successfully quitted smoking. Nevertheless, using biochemical validation is costly, difficult to obtain for all subjects, and may not correspond to the time frame of the assessment. Furthermore, the response rate for participating in biochemical validation is far less than self-report; this situation is not only in Hong Kong but also has been reported in elsewhere [29]. In fact, subjects might refuse to comply with a biochemical validation for a wide variety of reasons unrelated to smoking status, continuing to classify all these subjects as smokers would represent a distortion of the outcome data, resulting in a gross overestimate of smoking rates. After that, we decide to use self-reported 7-day point prevalence quit rate as the primary outcome.

## Conclusion

This study develops and validates practical smoking cessation interventions using risk communication approach to help cancer patients quit smoking and improve health related quality of life. It is anticipated that the findings can support the development of clinical evidence-based practice guidelines to promote smoking cessation in cancer patients. Most importantly, it will motivate more healthcare professionals to participate in helping cancer patients quit smoking.
